# A visible assay for evaluating the inhibitory activity of drug and antibody against HBV infection

**DOI:** 10.1128/spectrum.02638-25

**Published:** 2026-03-11

**Authors:** Hu Yan, Yuxiao Li, Congcong Liu, Huimin Guo, Yufeng Liu, Haiyan Wang, Xiangyang Ge, Zheng Zhang, Bin Ju

**Affiliations:** 1 Institute for Hepatology, National Clinical Research Center for Infectious Disease, Shenzhen Third People’s Hospital, The Second Affiliated Hospital, School of Medicine, Southern University of Science and Technology 255310, Shenzhen, Guangdong, China; 2 Guangdong Key Laboratory for Anti-infection Drug Quality Evaluation 554300 , Shenzhen, Guangdong, China; 3 Shenzhen Research Center for Communicable Disease Diagnosis and Treatment, Chinese Academy of Medical Sciences 624688, Shenzhen, Guangdong, China; Shandong First Medical University, Jinan, Shandong, China

**Keywords:** hepatitis B virus, visible immunospot assay, quantitative detection

## Abstract

Viral infectivity detection assays play an important role in the quantitative titers of virus and the effect of drugs and antibodies against virus infection. However, detecting the infectivity of hepatitis B virus (HBV) via conventional plaque assays still poses challenges because its infection does not result in cytopathic effects and progeny viruses struggle to infect adjacent cells *in vitro*. In this study, we established a sensitive and reliable immunospot assay for the visible quantitative detection of HBV infection based on an excellent detection antibody targeting the HBV core protein. Furthermore, this assay demonstrated robust performance in evaluating the inhibitory activity of antiviral drugs, as well as the neutralizing activity of monoclonal antibodies and plasma samples from vaccinated individuals against HBV infection. Collectively, our findings reported a sensitive and reliable method for the visible quantitative detection of HBV infectivity, offering significant potential for broad application in various HBV-related research fields.

**IMPORTANCE:**

In this study, we report a sensitive and reliable immunospot assay for the visible quantitative detection of HBV infection. This assay demonstrates excellent performance in evaluating the inhibitory effect of antiviral drugs, neutralizing antibodies, and plasma samples from vaccinated individuals. Given its robustness and versatility, the proposed method is anticipated to be an excellent alternative assay for HBV-related research, including therapeutic development and vaccine evaluation.

## INTRODUCTION

Virus detection and quantification methods are generally categorized into three groups: analyzing viral nucleic acid and protein, detecting viral infectivity, and directly enumerating physical viral particles. Although classical approaches, such as quantitative real-time PCR (Q-PCR) and enzyme-linked immunosorbent assay (ELISA), enable precise quantification of viral components, they are impossible to differentiate infectious virions from non-infectious or defective particles. Viral plaque assay and immuno-plaque assay are sensitive and reliable methods for quantifying viral infectivity, which are widely utilized for viral titration, antiviral drug evaluation, and neutralization assay (1, 2).

Hepatitis B virus (HBV) infection remains a significant global health burden. According to the Global Hepatitis Report 2024, an estimated 254 million individuals worldwide were living with chronic HBV infection in 2022, resulting in approximately 1.1 million deaths attributable to HBV-related complications (3). HBV, a partially double-stranded, relaxed circular DNA (rcDNA) virus, belongs to the family of Hepadnaviridae. The envelope contains three versions of HBV surface antigen (HBsAg)-large (L-HBsAg), middle (M-HBsAg), and small (S-HBsAg), which encapsulate the nucleocapsid assembled from core antigen (HBc) (4). For viral entry, the HBsAg loosely attaches to heparan sulfate proteoglycans (HSPGs) on the cell surface, and then pre-S1 interacts with its receptor sodium taurocholate cotransporting peptide (NTCP) with high affinity (5–7). Intracellularly, the rcDNA is transported to the nucleus and converted into covalently closed circular DNA (cccDNA), and then diverse mRNAs are transcribed and translated into different proteins, such as HBsAg, HBV e antigen (HBeAg), and HBc (8, 9). Secreted HBsAg and HBeAg are commonly used for assessing HBV infection status and antibody neutralization efficacy (10–12), which do not directly correlate with viral infectivity. In contrast, intracellular HBc antigen possesses the structural role and a regulatory function in most stages of the viral life cycle (13, 14), which serves as a critical biomarker for HBV infection. However, the non-cytopathic nature of HBV infection precludes the formation of visible plaques, which complicates the detection and quantification of infected cells using conventional plaque assay methodologies. Consequently, there is an urgent need to develop robust and standardized assays capable of quantitatively measuring HBV infectivity *in vitro*.

Previously, we reported a potent human HBc-specific monoclonal antibody (mAb), named cAbD4, which could sensitively recognize the individual HBV-infected cell by visible immunospot assay (15). Here, we further developed an enhanced immunospot assay utilizing the HRP-conjugated cAbD4, which demonstrated high sensitivity and reliability for the visible quantitative detection of HBV infection. This optimized assay system enables an excellent evaluation for both antiviral drug efficacy and antibody neutralization activity, thereby offering a valuable tool to advance HBV therapeutic development and pathogenesis research.

## MATERIALS AND METHODS

### Study approval and biological samples

The plasma samples were collected from five hepatitis B vaccine immunized individuals, YZ37, YZ48, YZ51, YZ78, and YZ116, with high anti-HBsAg antibody (HBsAb) titers. A control plasma with negative HBsAb titer was also involved in this study. All biological samples were stored in the Biobank of Shenzhen Third People’s Hospital and heat-inactivated at 56°C for 30 min before use. All individuals had provided the written informed consent for sample collection and subsequent analysis.

### Drugs, peptides, and antibodies

Myrcludex B lipopeptides (MGTNLSVPNPLGFFPDHQLDPAFGANSNNPDW-DFNPNKDHWPEANKVG), RG7834, and NVR 3-778 were purchased from MedChemExpress, and the control peptide (DTDFVNEFYAYLRKH) was synthesized by GenScript Biotech Corporation. Monoclonal antibodies (2H5-A14, H003, H015, and VRC01) were expressed and purified as previously described (15). All antibodies were quantified by NanoDrop and stored at −80°C before use. VRC01, an HIV-1-specific antibody, was used here as the negative control.

### Cell culture and virus infection

HBV particles were generated from HepAD38 and purified as previously described (15). Briefly, the supernatant of HepAD38 cells harboring HBV genome (MF967563.1) loaded onto a 15% sucrose cushion was centrifuged at 130,000 × *g* for 16 h at 4°C, and then the pellets containing virus were resuspended in Dulbecco’s Modified Eagle’s Medium (DMEM, Gibco).

HepG2-NTCP cells were cultured and infected with HBV as previously described (15). In brief, HepG2-NTCP cells were seeded into collagen-I-coated 96-well plates and infected with HBV at the indicated genome equivalent copies of HBV per cell (HBV gec/cell) in DMEM with 10% fetal bovine serum (FBS), 4% PEG8000, and 2.5% DMSO at 37°C for 24 h. Then, cells were washed with phosphate-buffered saline (PBS) and cultured in DMEM with 10% FBS, 1% penicillin/streptomycin, and 2.5% DMSO. The supernatants were collected every 2–3 days and detected by HBsAg ELISA and HBeAg ELISA kits according to the manufacturer’s instruction (Wantai Biological Pharmacy).

### Immunospot assay

Immunospot assay was conducted as previously described (15). Briefly, HepG2-NTCP cells were fixed with 4% paraformaldehyde and permeabilized with 0.1% Triton X-100 in PBS, each for 30 min at room temperature (RT). Subsequently, the cells were blocked with QuickBlock blocking buffer (Beyotime) for 1 h at RT, followed by overnight incubation with 0.25 μg/mL HRP-conjugated cAbD4 at 4°C. The enzymatic reaction was developed using KPL TrueBlue peroxidase substrates (Seracare Life Sciences), and HBV-infected cells were quantified using Cytation 7 Cell Imaging Multimode Reader (BioTek).

### Inhibition of drugs, antibodies, and plasma against HBV infection

HepG2-NTCP cells were incubated with Myrcludex B drug, which was serially threefold diluted from 100 nM at 37°C for 1 h and infected with 300 HBV gec/cell. As for RG7834 drugs, HepG2-NTCP cells were infected with 300 HBV gec/cell at 37°C for 24 h first, and then incubated with serially threefold diluted RG7834 from 100 nM. Antibodies (2H5-A14, H003, or H015) with serially fivefold diluted from 50 μg/mL and plasma samples with serially twofold diluted from 1:8 were mixed with 200 HBV gec/cell respectively at 37°C for 1 h. Then, the mixtures in the presence of 10% FBS, 4% PEG8000, and 2.5% DMSO were added into HepG2-NTCP cells for HBV infection. The supernatants were collected every 2–3 days and detected by HBeAg ELISA KIT according to the manufacturer’s instruction (Wantai Biological Pharmacy). The HBV-infected cells were identified by immunospot assay or immunofluorescence assay (IFA).

### Inhibition of NVR 3-778 against HBV infection

NVR 3-778 was serially twofold diluted from 12 μM and mixed with 360 HBV gec/cell in the presence of 10% FBS, 4% PEG8000, and 2.5% DMSO and added into HepG2-NTCP cells for HBV infection. NVR 3-778 drugs were replaced every 2–3 days. The supernatants were collected every 2–3 days and detected by HBeAg ELISA Kit according to the manufacturer’s instruction (Wantai Biological Pharmacy). The HBV-infected cells were identified by immunospot assay.

## RESULTS AND DISCUSSION

**Fig 1:**
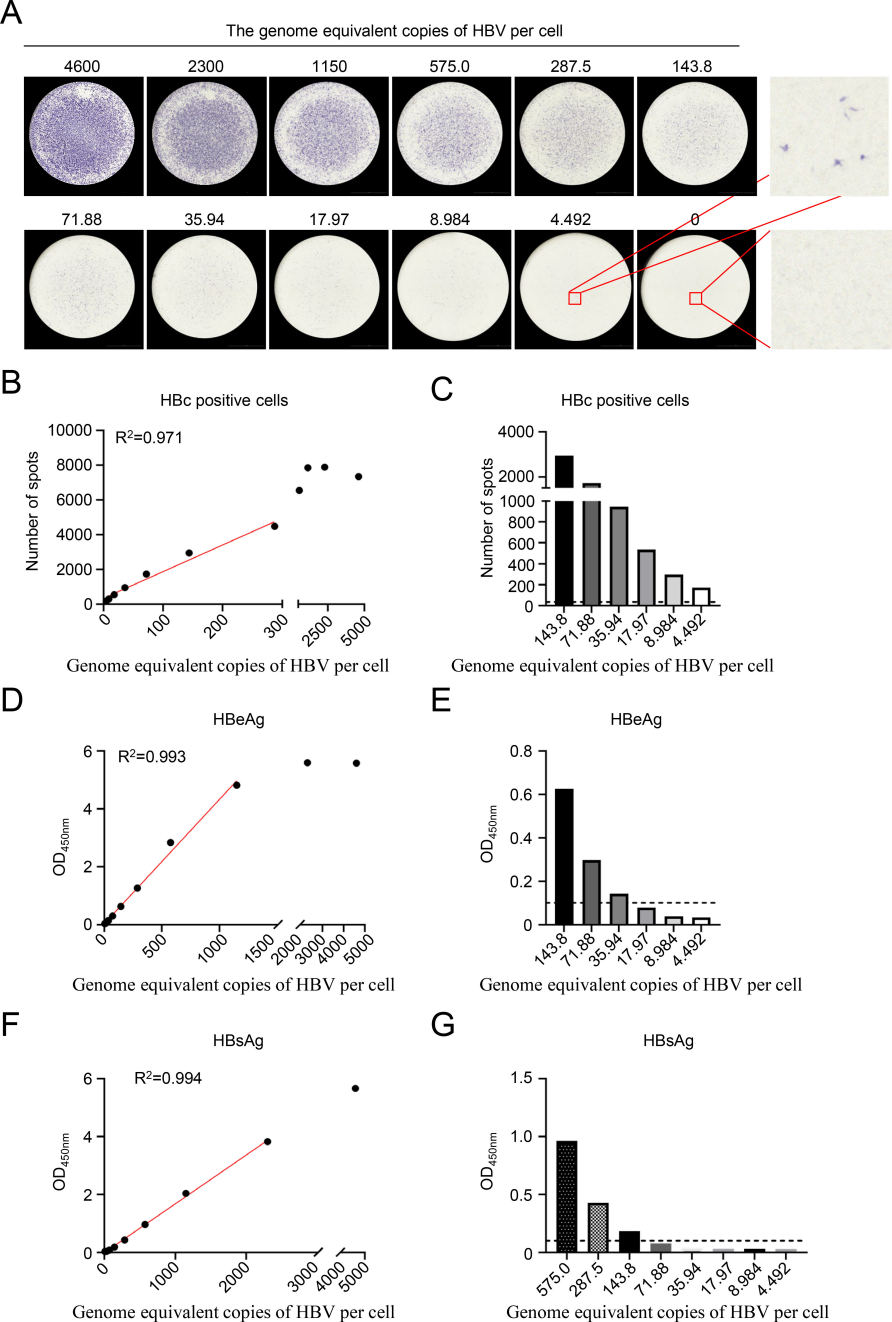
Establishment of an immunospot assay for the visible quantitative detection of HBV infection. HepG2-NTCP cells were infected with different genome equivalent copies of HBV per cell (HBV gec/cell). HBeAg and HBsAg at day 5 in supernatant was detected by ELISA, and the individual HBV-infected cell was detected by HRP-conjugated cAbD4 at 7 days after infection. (**A**) Immune spots of HBV-infected cells. (**B**) Correlation between the number of spots and HBV gec/cell. (**C**) Number of spots of certain HBV gec/cell infections. (**D**) Correlation between the HBeAg levels in supernatant and HBV gec/cell. (**E**) HBeAg OD_450nm_ of certain HBV gec/cell infections. (**F**) Correlation between the HBsAg levels in supernatant and HBV gec/cell. (**G**) HBsAg OD_450nm_ of certain HBV gec/cell infections. The dashed line indicates the detection limit of the assay. The experiment was independently performed at least twice, and one representative result was shown.

Previously, a potent human anti-HBc monoclonal antibody, named cAbD4, could effectively identify individual HBV-infected cells in an immunospot assay (15). To further investigate whether this assay is suitable for visible quantitative detection of HBV infection, HepG2-NTCP cells seeded into 96-well plates were infected with serially twofold diluted HBV. After 7 days, the HBV-infected cells were detected by HRP-conjugated cAbD4 and calculated by Cytation7. As expected, HRP-cAbD4 could clearly identify individual HBV-infected cells in a viral dose-dependent pattern (Fig. 1A). This immunospot assay achieved a linear detection for visible quantitative HBV infection within the range of 4.492 to 287.5 HBV gec/cell (Fig. 1B). Moreover, under the 4.492 HBV gec/cell infection, the HBV-infected cells were obviously identified (Fig. 1A and C). Compared with the ELISA detection of day 5 HBeAg in supernatant, the linear range was 35.94–1,150 HBV gec/cell with a lower limit of detection of 35.94 HBV gec/cell (Fig. 1D and E). Similarly, the linear range of the HBsAg detection was 143.8–2,300 HBV gec/cell with a lower limit of detection of 143.8 HBV gec/cell (Fig. 1F and G). Although HBeAg or HBsAg detection in supernatant could represent the high-titer HBV infection, their lower limits of detection were much higher than that of established visible immunospot assay. Since the OD_450nm_ of HBsAg detected in the supernatant is relatively lower than that of HBeAg, subsequent HBsAg analyses were not pursued. These results demonstrated that this immunospot assay enabled visible quantitative detection of HBV infection, even at relatively low viral titers (4.492 HBV gec/cell).

**Fig 2:**
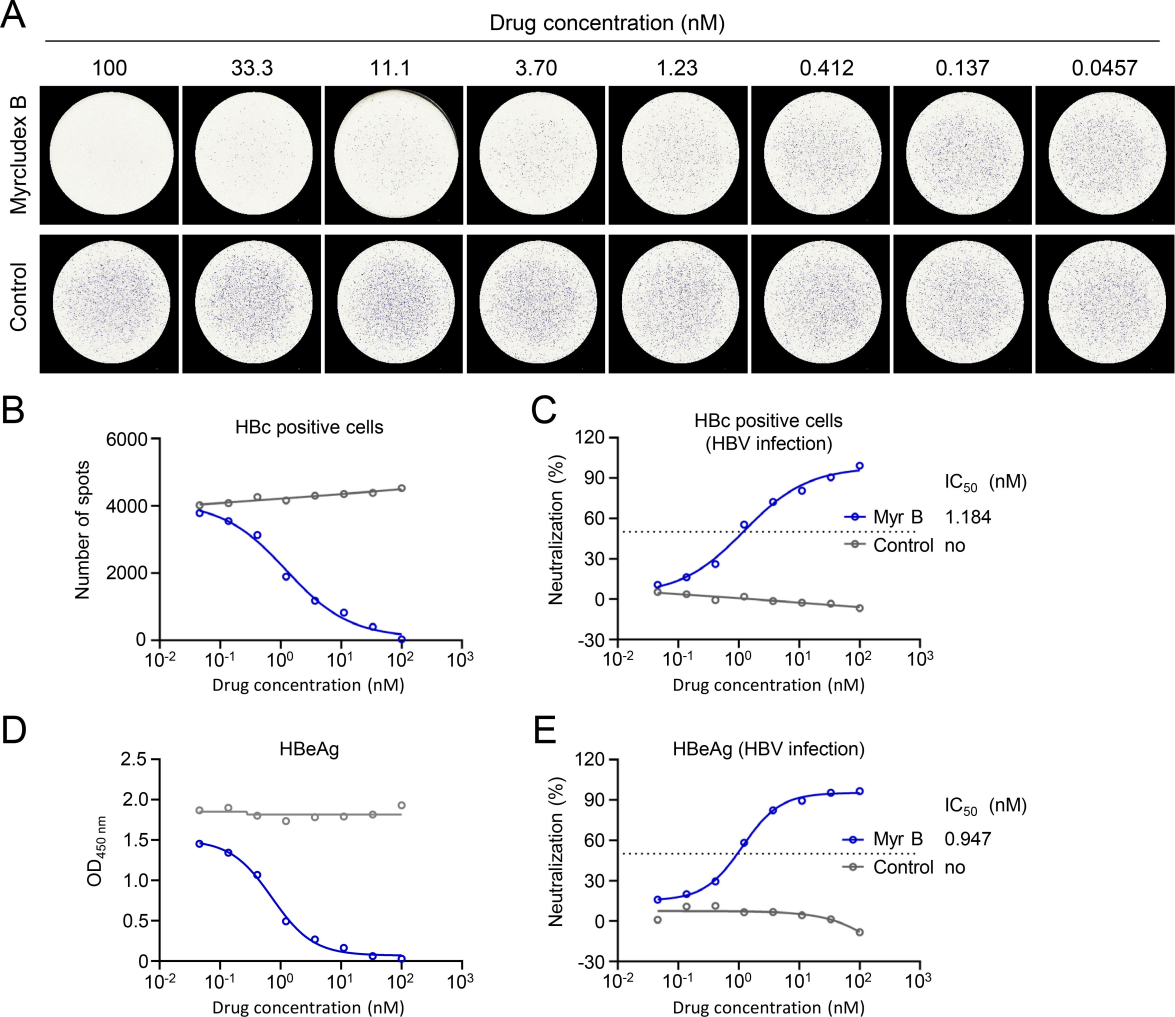
Evaluating the inhibitory efficacy of Myrcludex B against HBV infection by immunospot assay. HepG2-NTCP cells were incubated with Myrcludex B drugs at 37°C for 1 h, and then infected with 300 HBV gec/cell. After 7 days, individual HBV-infected cells were identified by HRP-conjugated cAbD4 (**A**), and the number of spots was calculated at different drug concentrations (**B**). (**C**) Inhibition curves of Myrcludex B against HBV infection from (**B**) were depicted. (**D**) Day 6 HBeAg in supernatant was detected by ELISA. (**E**) Inhibition curves of Myrcludex B against HBV infection from (**D**) were depicted. The dashed line indicates a 50% reduction in viral infectivity. The experiment was independently performed at least twice, and one representative result was shown.

**Fig 3:**
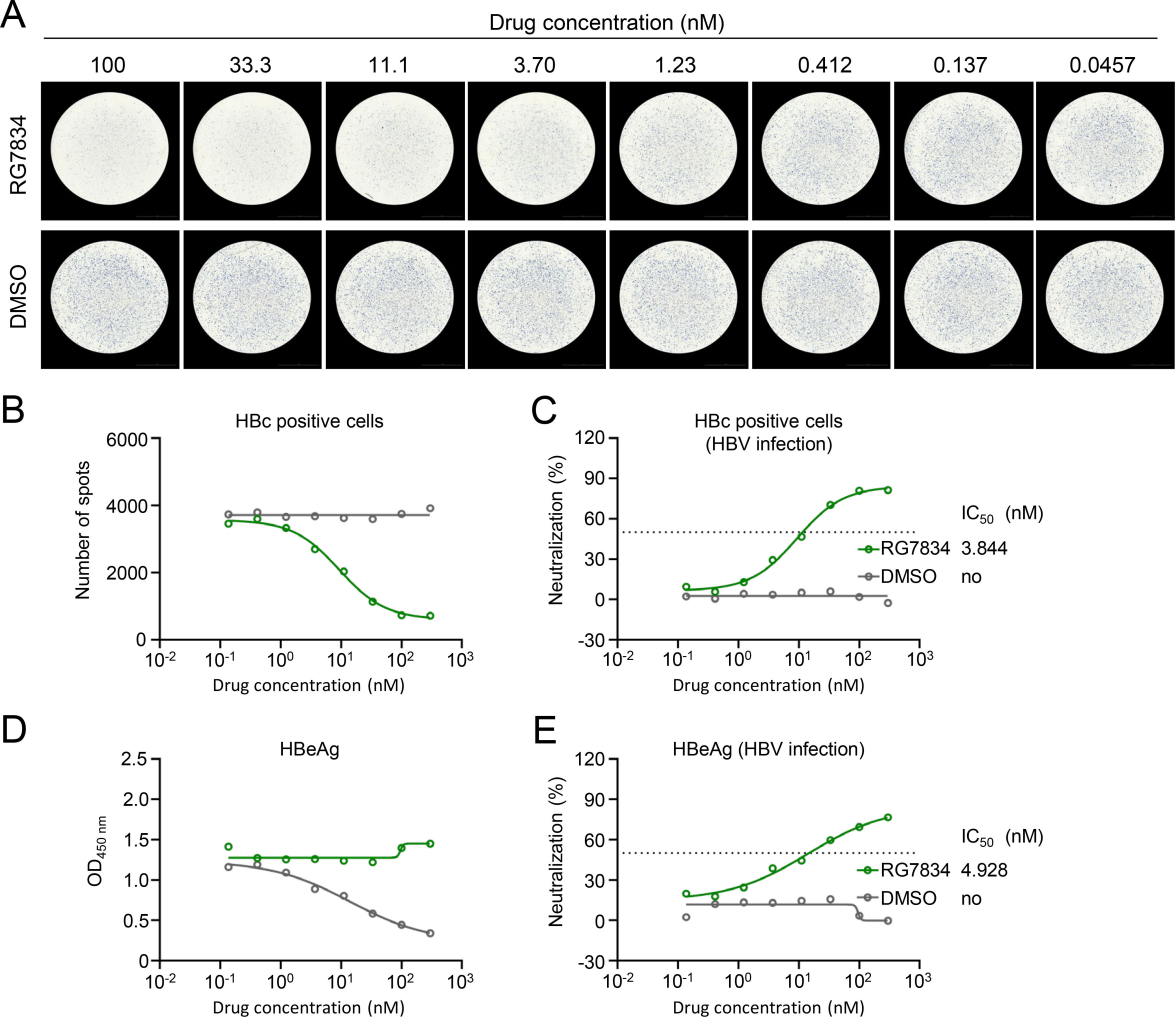
Evaluating the inhibitory efficacy of RG7834 drugs against HBV infection by immunospot assay. HepG2-NTCP cells were infected with 300 HBV gec/cell at 37°C for 24 h, and then incubated with RG7834 drugs. After 7 days, the individual HBV-infected cell was identified by HRP-conjugated cAbD4 (**A**) and calculated at different drug concentrations (**B**). (**C**) Inhibition curves of RG7834 against HBV infection from (**B**) were depicted. (**D**) HBeAg in supernatant from day 3 to 6 was detected by ELISA. (**E**) Inhibition curves of RG7834 against HBV infection from (**D**) were depicted. The dashed line indicates a 50% reduction in viral infectivity. The experiment was independently performed at least twice, and one representative result was shown.

To further demonstrate the utility of this visible immunospot assay, we evaluated its application in assessing the inhibitory effects of antiviral drugs on HBV infection and replication. We first tested Myrcludex B, a 47-amino acid peptide derived from the pre-S1 domain of HBV known to effectively inhibit HBV infection (16–18). Compared with the control group, HBV-infected spots detected by HRP-cAbD4 significantly reduced with the increasing concentration of Myrcludex B (Fig. 2A and B). The 50% inhibition concentration (IC_50_) value for Myrcludex B was determined to be 1.184 nM (Fig. 2C). This inhibitory trend was corroborated by ELISA analysis of HBeAg in supernatant, which yielded a similar IC_50_ value of 0.947 nM for Myrcludex B (Fig. 2D and E). We further employed the immunospot assay to evaluate RG7834, another antiviral agent. RG7834, a dihydroquinolizinone compound, potently suppresses HBsAg and HBeAg expressions by destabilizing multiple HBV mRNA species during viral replication (17, 18). The immunospot assay detected an IC_50_ value of 3.844 nM for RG7834 (Fig. 3A through C), which was consistent with the IC_50_ value of 4.928 nM derived from HBeAg suppression assay (Fig. 3D and E). Finally, we evaluated the antiviral activity of NVR 3-778, a capsid assembly modulator (CAM) reported to inhibit DNA replication by accelerating their assembly process and preventing the formation of cccDNA in de novo-infected primary human hepatocytes (19, 20). Using this immunospot assay, the IC_50_ value of NVR 3-778 was calculated as 9.441 μM ( Fig. S1A through C), which was similar to the IC_50_ value of 2.700 μM obtained from HBeAg suppression assay (Fig. S1D and E). Although the IC_50_ value measured in this study differed considerably from previously reported values based on HBV-DNA detection, it overall aligned with the value obtained from HBeAg assay (20). This suggests that this immunospot assay can effectively evaluate the inhibitory efficacy of NVR 3-778 against HBV in *de novo*-infected cells. Collectively, these results indicated that this established immunospot assay detected by HRP-cAbD4 was excellently applied in the evaluation for the efficacy of HBV-targeting antiviral drugs.

**Fig 4:**
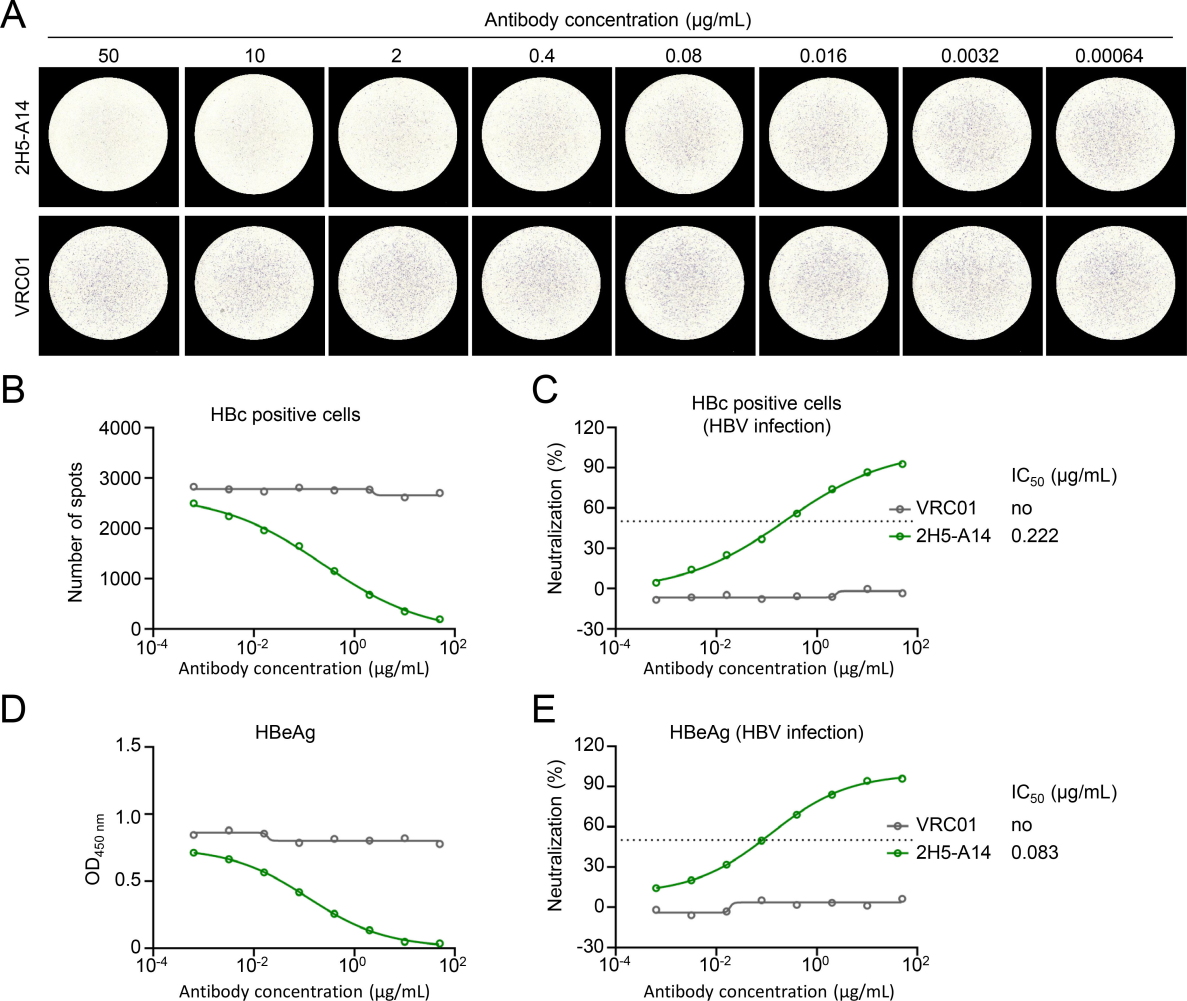
Evaluating the neutralizing activity of pre-S1 antibody by immunospot assay. A pre-S1 antibody 2H5-A14 and the control antibody VRC01 were serially fivefold diluted from 50 μg/mL, and then incubated with 200 HBV gec/cell respectively at 37°C for 1 h. Subsequently, the antibody-virus mixtures were added to HepG2-NTCP cells. After 7 days, individual HBV-infected cells were identified by HRP-conjugated cAbD4 (**A**), and the number of spots was calculated at different antibody concentrations (**B**). (**C**) Neutralization activity against HBV from (**B**) was analyzed. (**D**) Day 6 HBeAg in supernatant was detected by ELISA. (**E**) Neutralization activity against HBV from (**D**) was analyzed. VRC01 is an HIV-1 antibody, as negative control. The dashed line indicates a 50% reduction in viral infectivity. Each experiment was independently performed at least twice, and one representative result was shown.

**Fig 5:**
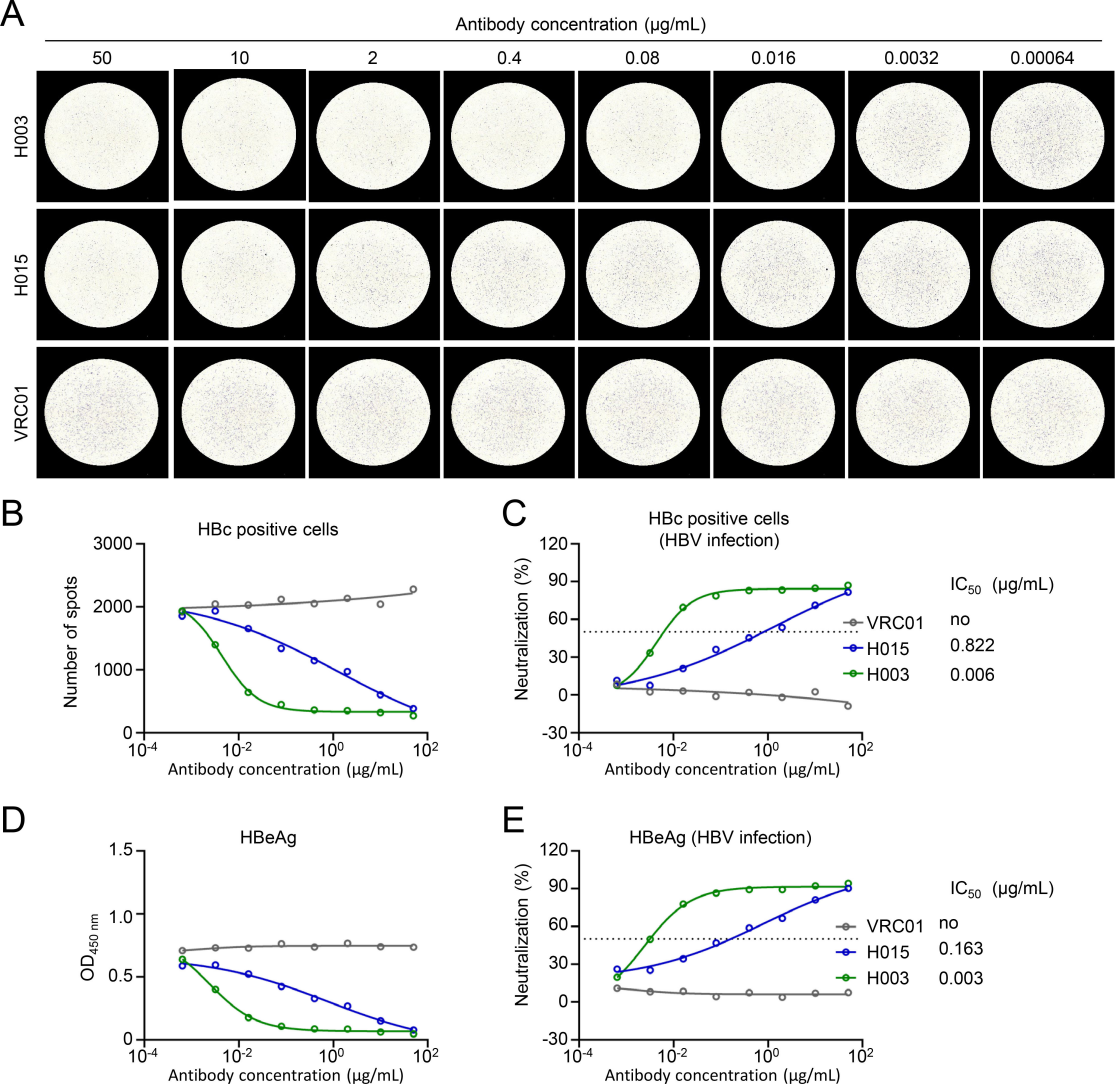
Evaluating the neutralizing activity of anti-S-HBsAg antibody by immunospot assay. Two anti-S-HBsAg antibodies H003, H015, and the control antibody VRC01 were serially fivefold diluted from 50 μg/mL, then incubated with 200 HBV gec/cell at 37°C for 1 h. Subsequently, the antibody-virus mixtures were added to HepG2-NTCP cells. After 7 days, HBV-infected cells were detected by immunospot assay (**A**), and the individual HBV-infected cell was calculated at different antibody concentrations (**B**). (**C**) Neutralization activity of H003 and H015 against HBV from (**B**) was analyzed. (**D**) Day 6 HBeAg in supernatant was detected by ELISA. (**E**) Neutralization activity against HBV from (**D**) was analyzed. VRC01 is an HIV-1 antibody, as negative control. The dashed line indicates a 50% reduction in viral infectivity. Each experiment was independently performed at least twice, and one representative result was shown.

Neutralizing antibodies play a vital role in suppressing HBV infection. Anti-preS1 antibody can block the recognition of HBV to its receptor NTCP, while anti-HBsAg antibody can inhibit the attachment of virions to HSPGs on the cell surface (12, 21). Currently, the neutralization of these antibodies was primarily evaluated by indirectly detecting the HBeAg or HBsAg *in vitro* assay. In order to evaluate the neutralization of antibodies by focus reduction neutralization test, HepaG2-NTCP cells were infected with 300 HBV gec/cell pre-incubated with serially fivefold diluted pre-S1 antibody 2H5-A14 (21) and detected by immunospot assay. Compared with an HIV-1 antibody as the irrelevant control antibody, the HBV-infected cells decreased with the increasing concentration of 2H5-A14 (Fig. 4A and B), the IC_50_ of which by immunospot assay detection was calculated as 0.222 μg/mL (Fig. 4C). By contrast, HBeAg in the supernatant also decreased with the increasing concentration of 2H5-A14. The IC_50_ of 2H5-A14 detected by HBeAg was 0.083 μg/mL, which is nearly the IC_50_ detected by immunospot assay (Fig. 4D and E). Next, the neutralization of two reported anti-S-HBsAg antibodies H003 and H015 (11) was also evaluated. Both the HBV-infected spots and HBeAg levels in supernatant decreased with the increasing concentrations of H003 or H015 (Fig. 5A, B and D). The IC_50_ of H003 detected by immunospot assay was 0.006 μg/mL, which was similar to that analyzed by HBeAg detection (IC_50_ = 0.003 µg/mL) (Fig. 5C and E). Meanwhile, the IC_50_ of H015 detected by immunospot assay was 0.822 μg/mL, slightly higher than that evaluated by HBeAg detection (IC_50_ = 0.163 µg/mL) (Fig. 5C and E). Such variations in IC_50_ values between different assays were also observed by other groups, which showed IC_50_ values detected by immunofluorescence assay were higher than that evaluated by HBeAg detection (11). In conclusion, we established an excellent visible immunospot assay to evaluate the neutralization of monoclonal antibodies to inhibit HBV infection.

**Fig 6:**
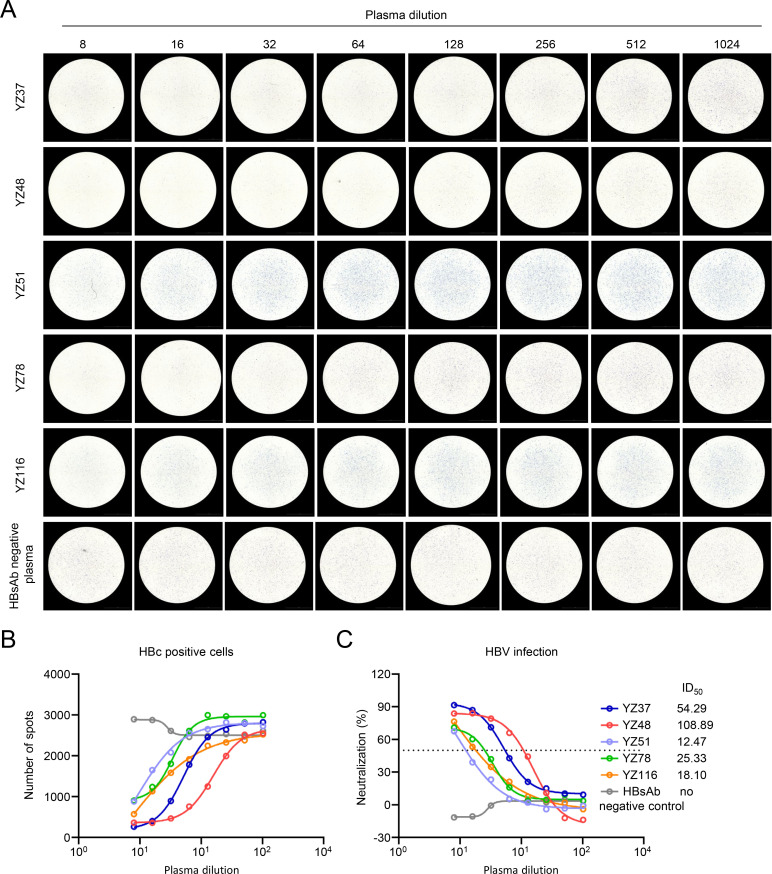
Evaluating the neutralizing activity of plasma polyclonal antibodies by immunospot assay. All samples were serially twofold diluted from 1:8 and mixed with 200 HBV gec/cell respectively at 37°C for 1 h. Then, the mixtures were added into HepG2-NTCP cells. After 7 days, individual HBV-infected cells were identified by HRP-conjugated cAbD4 in immunospot assay (**A**), and the number of spots was calculated at different plasma dilution (**B**). (**C**) The neutralizing activity (ID_50_) of plasma was analyzed from (**B**). The dashed line indicates a 50% reduction in viral infectivity. The experiment was independently performed at least twice, and one representative result was shown.

To further investigate the potential application of this visible immunospot assay, the neutralization of five plasma samples with high HBsAb titers from HBV vaccinees was evaluated under 200 HBV gec/cell infection. HBV-infected cells increased to varying extents with the increasing dilution of these five plasma samples (Fig. 6A and B). In contrast, the control plasma with negative HBsAb titer showed no inhibitory effect (Fig. 6A and B). Moreover, the 50% inhibitory dilution (ID_50_) of these five plasma samples, with a range from 12.47 to 108.89, was successfully calculated (Fig. 6C). These results indicated this established visible immunospot assay could evaluate the neutralization of plasma polyclonal antibodies.

In this study, we developed a highly sensitive immunospot assay for the visible quantitative detection of HBV infection. IFA targeting HBc-specific cells has also been employed for HBV detection. In assessing the inhibitory activity of Myrcludex B test, the IFA-derived IC_50_ (2.908 nM) was comparable to that obtained via immunospot assay (1.184 nM). Nevertheless, this IFA exhibited non-specific background signals in 96-well plates (Fig. S2). Both IFA and immunospot assay necessitate specialized instrument to detect the HBc-specific cells. IFA requires fluorescence microscopy, whereas immunospot assay utilizes brightfield microscopy, making immunospot assay relatively more accessible in most laboratories. The immunospot assay demonstrated high efficacy in evaluating the inhibitory effects of antiviral drugs, monoclonal antibodies, and polyclonal antibodies against HBV infection, exhibiting excellent reproducibility. Although HBeAg or HBsAg levels are conventional markers for HBV infection, neutralization potencies of certain antibodies (e.g., H015) measured by HBeAg/HBsAg detection may be inconsistent with that derived from HBc-positive cells detection, which has also been reported by another study (11). A lower IC_50_ value in one assay does not invariably indicate more superior efficacy, as IC_50_ measurements can vary depending on the assay system. The immunospot assay has been widely used for assessing neutralizing antibodies against other viruses, including SARS-CoV-2 and Rift Valley fever virus (22, 23). Compared with HBeAg or HBsAg detection, the HBc-targeted immunospot assay provides a more direct relevant assessment of inhibitory effects against HBV infection. In addition, the amino acids E77 and D78 of HBc have been identified as key binding sites for cAbD4, which can also recognize the intracellular HBeAg precursor; however, this cross-reactivity does not compromise the validity of our findings. Collectively, our results demonstrated a sensitive and robust immunospot assay for visible quantitative detection of HBV infection, making it suitable for viral titration, drug efficacy assessment, and neutralization studies. This visible immunospot assay will also provide a convenient way to evaluate plasma neutralization response in clinical trials for chronic hepatitis B functional cure.

## Data Availability

This paper does not include any high-throughput sequencing or other large data sets. This paper does not report any original code. Any additional information required to reanalyze the data reported in this paper are available from the corresponding author upon request.

## SUPPLEMENTAL MATERIAL

Supplemental material spectrum.02638-25-s0001.pdf Fig. S1 and S2.

ASM does not own the copyrights to Supplemental Material that may be linked to, or accessed through, an article. The authors have granted ASM a non-exclusive, world-wide license to publish the Supplemental Material files. Please contact the corresponding author directly for reuse.
