# Supplementary material for: A visible assay for evaluating the inhibitory activity of drug and antibody against HBV infection
Source: Microbiol Spectr. 2026 Mar 11;14(4):e02638-25. doi: 10.1128/spectrum.02638-25 (PMC13055246; doi:10.1128/spectrum.02638-25)
Supplement: Supplemental material — Fig. S1 and S2. [file spectrum.02638-25-s0001.pdf]

Supplementary information

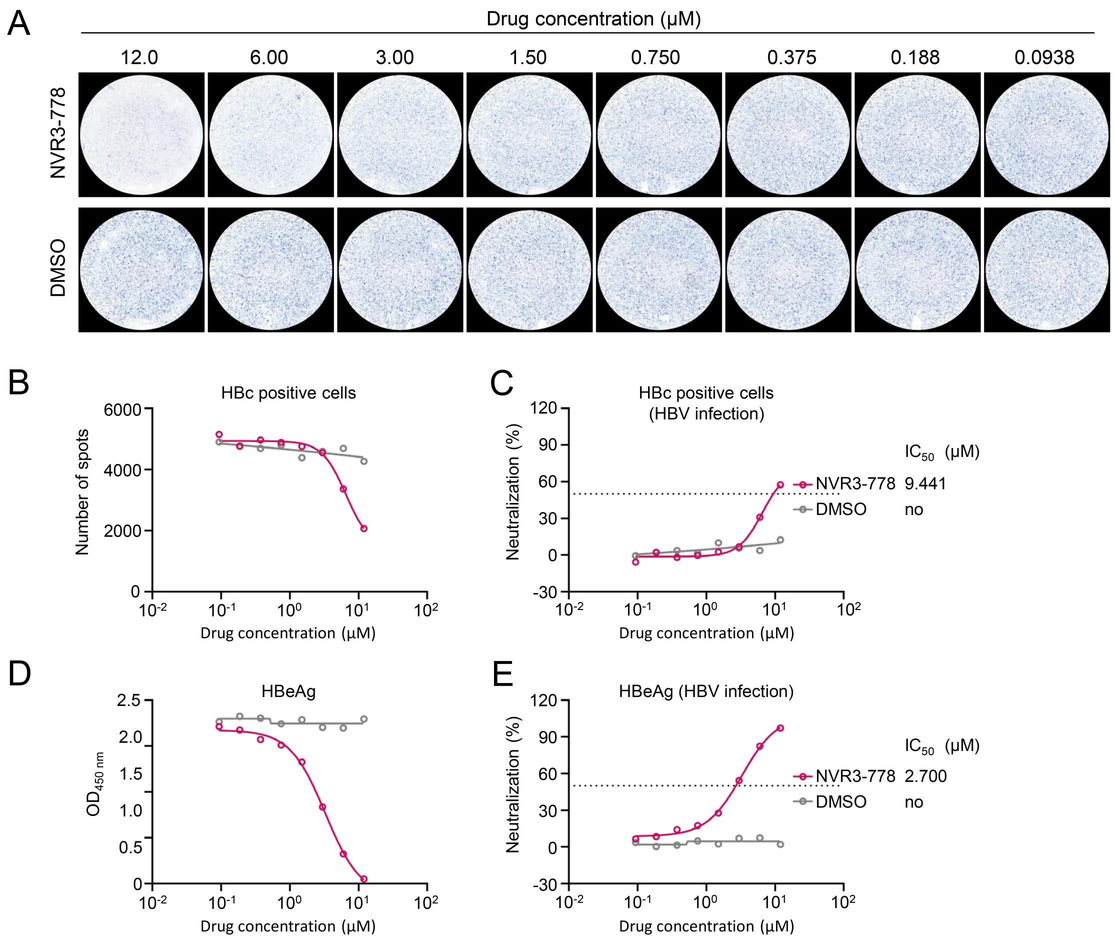

Figure S1. Evaluating the inhibitory efficacy of NVR 3-778 drugs against HBV infection by immunospot assay.

HepG2-NTCP cells were infected with 360 HBV gec/cell coupled with different concentrations of NVR 3-778 drugs at 37°C for 24 hours. The drugs were replaced every 2-3 days. After 7 days, the individual HBV-infected cell was identified by HRP-conjugated cAbD4 (**A**) and calculated at different drug concentrations (**B**). (**C**) Inhibition curves of NVR 3-778 against HBV infection from (B) was depicted. (**D**) HBeAg in supernatant from day 3 to day 6 was detected by ELISA. (**E**) Inhibition curves of NVR 3-778 against HBV infection from (D) was depicted. The dashed line indicates a 50% reduction in viral infectivity. The experiment was independently performed at least twice and one representative result was shown.

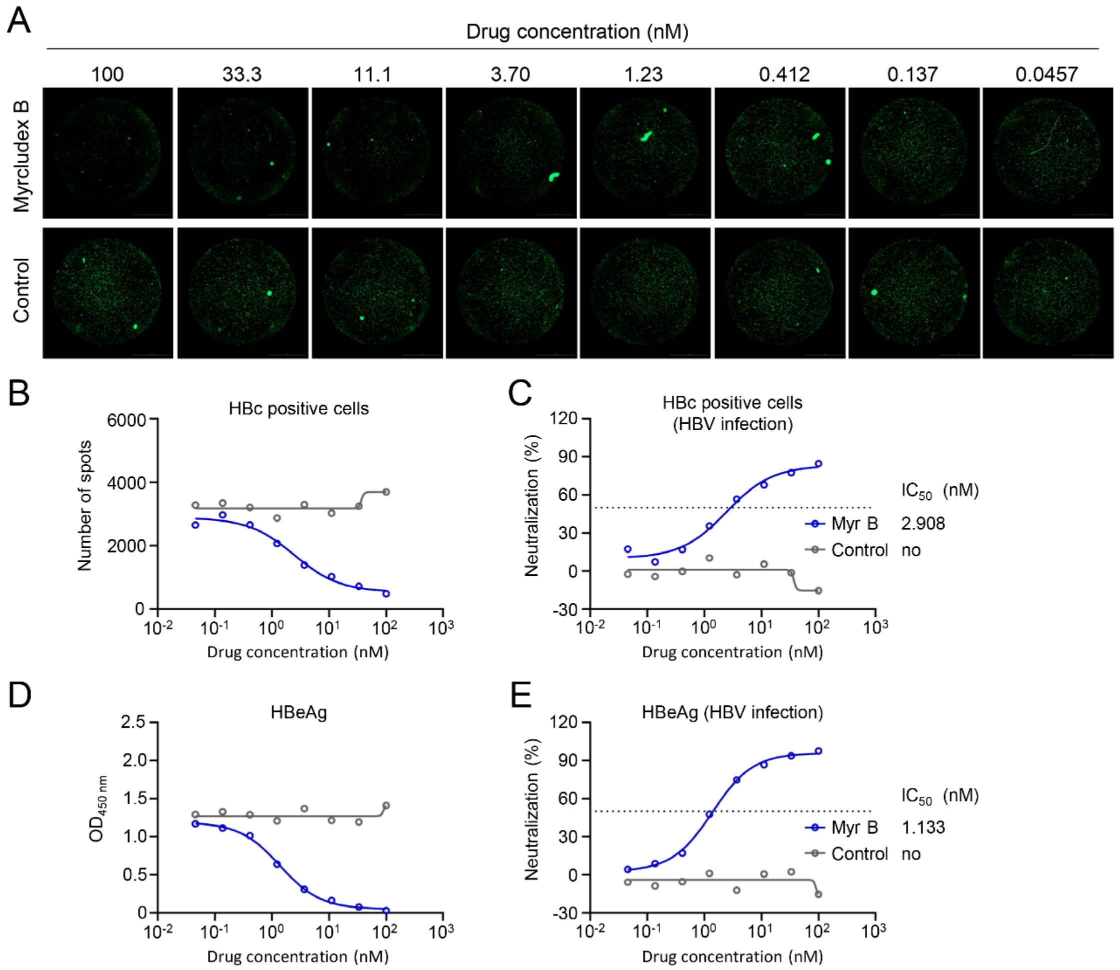

Figure S2. Evaluating the inhibitory efficacy of Myrcludex B against HBV infection by immunofluorescence assay.

HepG2-NTCP cells were incubated with Myrcludex B drugs at 37°C for 1 hour, and then infected with 300 HBV gec/cell. After 7 days, the individual HBV-infected cell was identified by cAbD4 with goat anti-Human IgG Alexa Flour^{TM} 488 (**A**) and calculated at different drug concentrations (**B**). (**C**) Inhibition curves of Myrcludex B against HBV infection from (B) was depicted. (**D**) HBeAg in supernatant from day 3 to day 6 was detected by ELISA. (**E**) Inhibition curves of Myrcludex B against HBV infection from (D) was depicted. The dashed line indicates a 50% reduction in viral infectivity. The experiment was independently performed at least twice and one representative result was shown.
